# REV-ERBα and REV-ERBβ function as key factors regulating Mammalian Circadian Output

**DOI:** 10.1038/s41598-019-46656-0

**Published:** 2019-07-15

**Authors:** Ryosuke Ikeda, Yoshiki Tsuchiya, Nobuya Koike, Yasuhiro Umemura, Hitoshi Inokawa, Ryutaro Ono, Maho Inoue, Yuh Sasawaki, Tess Grieten, Naoki Okubo, Kazuya Ikoma, Hiroyoshi Fujiwara, Toshikazu Kubo, Kazuhiro Yagita

**Affiliations:** 1 Department of Physiology and Systems Bioscience, Kyoto Prefectural University of Medicine, Kawaramachi-Hirokoji, Kamigyo-ku, Kyoto 602-8566 Japan; 2 Department of Orthopaedics, Kyoto Prefectural University of Medicine, Kawaramachi-Hirokoji, Kamigyo-ku, Kyoto 602-8566 Japan

**Keywords:** Circadian rhythms, Gene expression

## Abstract

The circadian clock regulates behavioural and physiological processes in a 24-h cycle. The nuclear receptors REV-ERBα and REV-ERBβ are involved in the cell-autonomous circadian transcriptional/translational feedback loops as transcriptional repressors. A number of studies have also demonstrated a pivotal role of REV-ERBs in regulation of metabolic, neuronal, and inflammatory functions including bile acid metabolism, lipid metabolism, and production of inflammatory cytokines. Given the multifunctional role of REV-ERBs, it is important to elucidate the mechanism through which REV-ERBs exert their functions. To this end, we established a *Rev-erbα*/*Rev-erbβ* double-knockout mouse embryonic stem (ES) cell model and analyzed the circadian clock and clock-controlled output gene expressions. A comprehensive mRNA-seq analysis revealed that the double knockout of both *Rev-erbα* and *Rev-erbβ* does not abrogate expression rhythms of E-box-regulated core clock genes but drastically changes a diverse set of other rhythmically-expressed output genes. Of note, REV-ERBα/*β* deficiency does not compromise circadian expression rhythms of PER2, while REV-ERB target genes, *Bmal1* and *Npas2*, are significantly upregulated. This study highlight the relevance of REV-ERBs as pivotal output mediators of the mammalian circadian clock.

## Introduction

The circadian clock is an endogenous biological clock with a period of about 24 h and regulates diverse behavioral and physiological functions. In mammals, the master circadian pacemaker resides in the suprachiasmatic nucleus (SCN) of the hypothalamus and coordinates other circadian oscillators that exist in most peripheral tissues throughout the body^1–3^. It is widely accepted that a set of transcription factors comprise cell-autonomous transcriptional and translational feedback loops that enable circadian oscillation of gene expression^4,5^. In the primary loop, transcription factors BMAL1 and CLOCK heterodimerize and promote transcription of *Period* genes (*Per1*, *Per2*, *Per3*) and *Cryptochrome* genes (*Cry1* and *Cry2*). The PER and CRY proteins translocate into the nucleus and inhibit transcriptional activity of CLOCK/BMAL1 heterodimer and thereby repress their own expression. In addition, *Bmal1* expression is regulated by the secondary feedback loop, in which CLOCK and BMAL1 induce *Rev-erbα* (*Nr1d1*) and *Rev-erbβ* (*Nr1d2*) expression and then REV-ERBα/REV-ERBβ proteins repress *Bmal1* (*Arntl*) expression by competing bindings of transcriptional activators, RORα and RORγ, to the ROR-response element (RRE) in the *Bmal1* promoter. Thus, REV-ERBs are thought to be key regulators of the RRE-mediated transcriptional oscillation^6,7^. It has been reported that *Rev-erbα* knockout in mouse results in a relatively mild effect on circadian behavioral rhythms^6^. It has also been reported that liver-specific deficiency and the induced deficiency of both *Rev-erbα* and *Rev-erbβ* in adult mice results in altered circadian gene expressions and disrupted behavioral rhythms, respectively^8^. These observations suggest the redundant and essential role of REV-ERBα and REV-ERBβ in circadian clock regulation. However, the lacking conventional double knockout of *Rev-erbα*/*β* due to the lethality during development makes it difficult to know whether REV-ERBs are necessary and universal factors for ticking of the cell-autonomous circadian clockwork. REV-ERBs are also known to regulate the expression of multiple downstream genes involved in diverse cellular functions including metabolism and inflammation^9–14^. In liver, REV-ERBs regulate a number of genes involved in metabolic pathways in collaboration with HNF6^15^. In macrophages, REV-ERBs regulate downstream genes including *Cx3cr1* and *Mmp9* by repressing enhancer RNA expression^16^. While accumulating evidence suggests the importance of REV-ERBs in regulation of a wide range of cellular physiology, it still remains to be elucidated how REV-ERBs regulate a different set of downstream genes in a cell type-specific manner. In this study, we established the *Rev-erbα*/*Rev-erbβ* double knockout mouse embryonic stem (mES) cell line and examined the effect of REV-ERBs deficiency on the cell-autonomous circadian clockwork as well as global changes of circadian gene expression rhythms. These analyses emphasize REV-ERBs function to form an essential link between the circadian clock and a wide variety of output gene expression rhythms.

## Results

### Establishment of REV-ERBα/β-deficient mES cells

**Figure 1:**
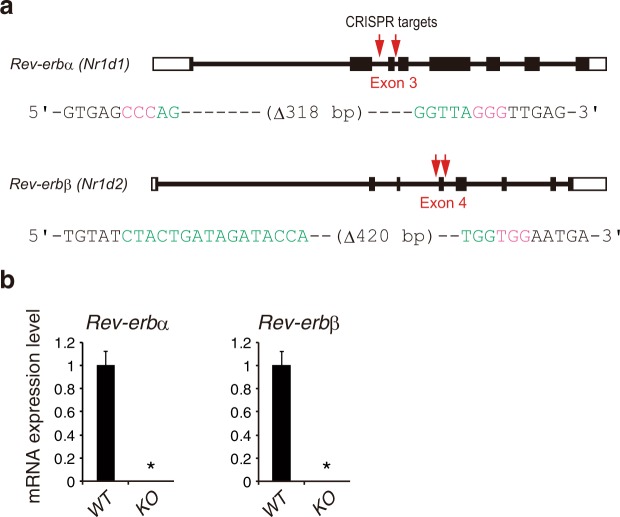
CRISPR/Cas9-mediated targeting of *Rev-erbα* and *Rev-erbβ* in mES cells. (**a**) Schematic of *Rev-erbα* and *Rev-erbβ* target regions. Green and pink letters indicate the CRISPR-targeted sequence and the PAM sequence, respectively (see Methods). (**b**) Relative expression levels of *Rev-erbα* and *Rev-erbβ* mRNA including CRISPR-targeted exon were determined by quantitative PCR. The values were normalized to *18S rRNA* and presented as means ± SD (n = 3; *p < 0.001).

To investigate the role of REV-ERBs in the cell-autonomous circadian gene expression, we employed the ES cell-differentiation assay that enables reproducible formation of the circadian rhythms of clock gene expression^17,18^. We utilized *Per2::Luciferase* (*Per2*^*Luc*^) knock-in mES cells^19–21^ and introduced the targeted deletions of exons of *Rev-erbα* and *Rev-erbβ* genes by using CRISPR/Cas9 systems (Fig. 1a). In knockout cells, translational frameshifts were confirmed by sequencing of cDNA (Supplementary Fig. S1) and mRNAs including the CRISPR-targeted exon were not detected by quantitative PCR (Fig. 1b), indicating successful establishment of *Rev-erbα*/*Rev-erbβ* double knockout mES cells.

### Global gene expression analysis in differentiated REV-ERBα/β-deficient mES cells

**Figure 2:**
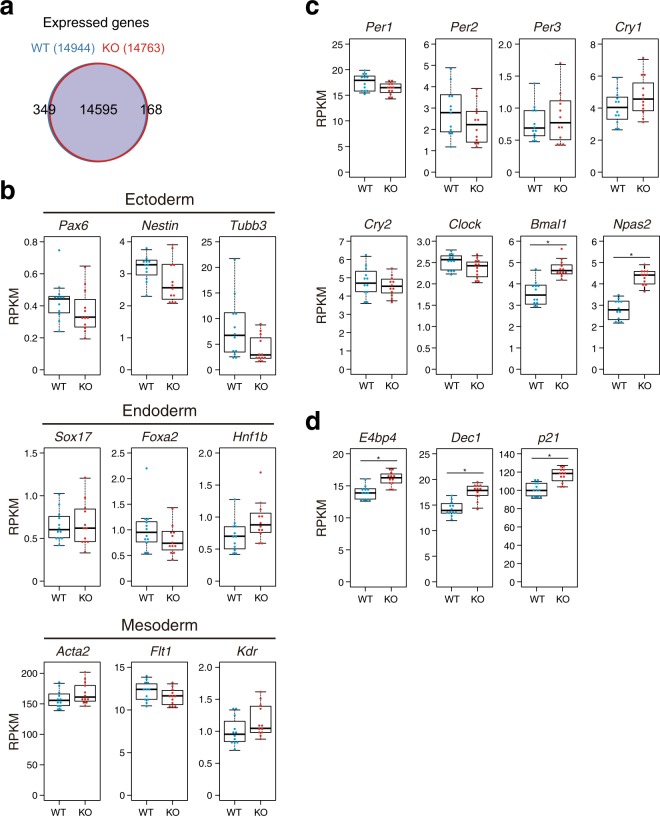
REV-ERBα/β deficiency affects their target gene expression in differentiated mES cells. (**a**) Venn diagram of expressed genes in WT and KO cells. (**b**) Expression of specific differentiation markers of the three germ layers. RPKM values from 12 time points are shown in bee swarm box plots. (**c**) Expression of core clock genes (n = 12; *p < 0.01). (**d**) Expression of known target genes of REV-ERBα/β (n = 12; *p < 0.01).

To evaluate the impact of REV-ERBs deficiency on global gene expression, the cells were differentiated with the embryoid body (EB) formation method and temporal RNA sequencing (RNA-seq) analysis with RNA samples at 4-h intervals over 2 days were performed. RNA samples from control *Per2*^*Luc*^ cells (WT) and *Rev-erbα*/*β* deficient cells (KO) were analyzed by polyA-selected RNA-seq. The number of expressed genes in WT and KO cells were comparable and most of them overlapped (Fig. 2a). Expression levels of several differentiation marker genes for ectoderm, endoderm, and mesoderm were not drastically changed between WT and KO cells, suggesting that overall differentiation of *Rev-erbα*/*β* deficient cells is likely to be comparable to that of WT cells (Fig. 2b). We evaluated the mRNA expression of core clock genes and found that expression levels of *Per1*, *Per2*, *Per3*, *Cry1*, *Cry2*, and *Clock* in *Rev-erbα*/*β* deficient cells were comparable to those in WT (Fig. 2c). In contrast, the expression levels of *Bmal1* and *Npas2*, the direct targets of REV-ERBα/β, were significantly upregulated (Fig. 2c). Furthermore, previously reported REV-ERB-target genes such as *E4bp4* (*Nfil3*), *Dec1* (*bhlhe40*), and *p21* (*Cdkn1a*)^22–24^ were also upregulated (Fig. 2d). These results indicate that REV-ERBα/β deficiency in differentiated mES cells affects their target gene expression in a manner consistent with REV-ERBs function as transcriptional repressors.

### REV-ERB deficiency alters diverse gene expression rhythms in differentiated mES cells

**Figure 3:**
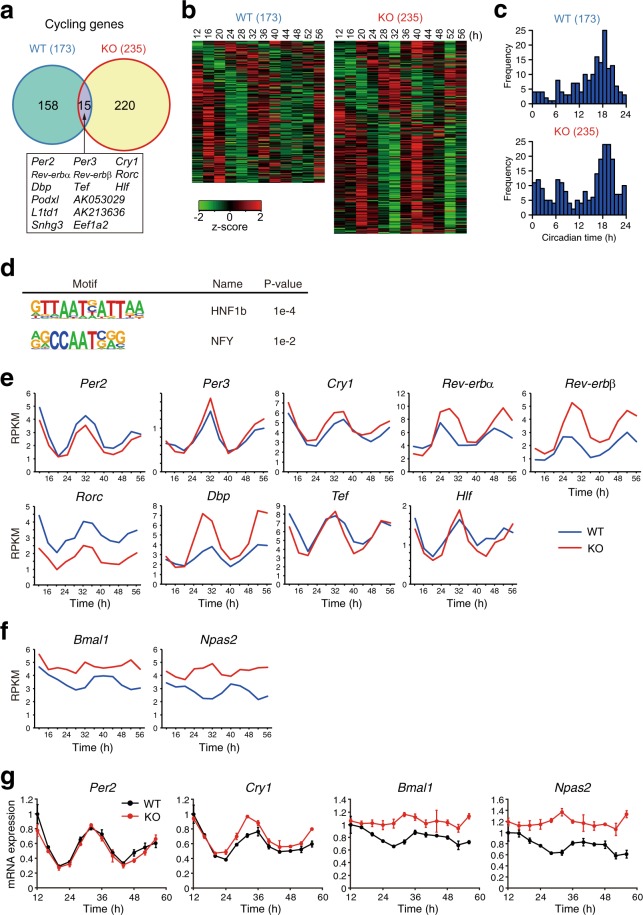
Comprehensive analysis of circadian gene expression in *Rev-erbα/β*-deficient cells. (**a**) Venn diagram of cycling genes in WT and KO cells. (**b**) Heatmap view of cycling genes. Each gene is represented as a horizontal line ordered vertically by phase as determined by MetaCycle. (**c**) The phase distribution of cycling genes. (**d**) Homer known motif enrichment analysis reveals HNF1b and NF-Y binding motifs are enriched in promoter region of cycling genes in WT cells but not in KO cells. (**e**) Expression of circadian clock genes cycling in both WT and KO cells. mRNA expression levels in WT and KO cells are plotted with blue and red lines, respectively. (**f**) Expression levels of *Bmal1* and *Npas2* in WT and KO cells are plotted with blue and red lines, respectively. (**g**) Cyclic expression of *Per2*, *Cry1*, *Bmal1*, and *Npas2* is analyzed by quantitative PCR. mRNA expression levels in WT and KO cells are plotted with black and red lines, respectively. The values were normalized to *18S rRNA* and presented as means ± SD (n = 3).

**Figure 4:**
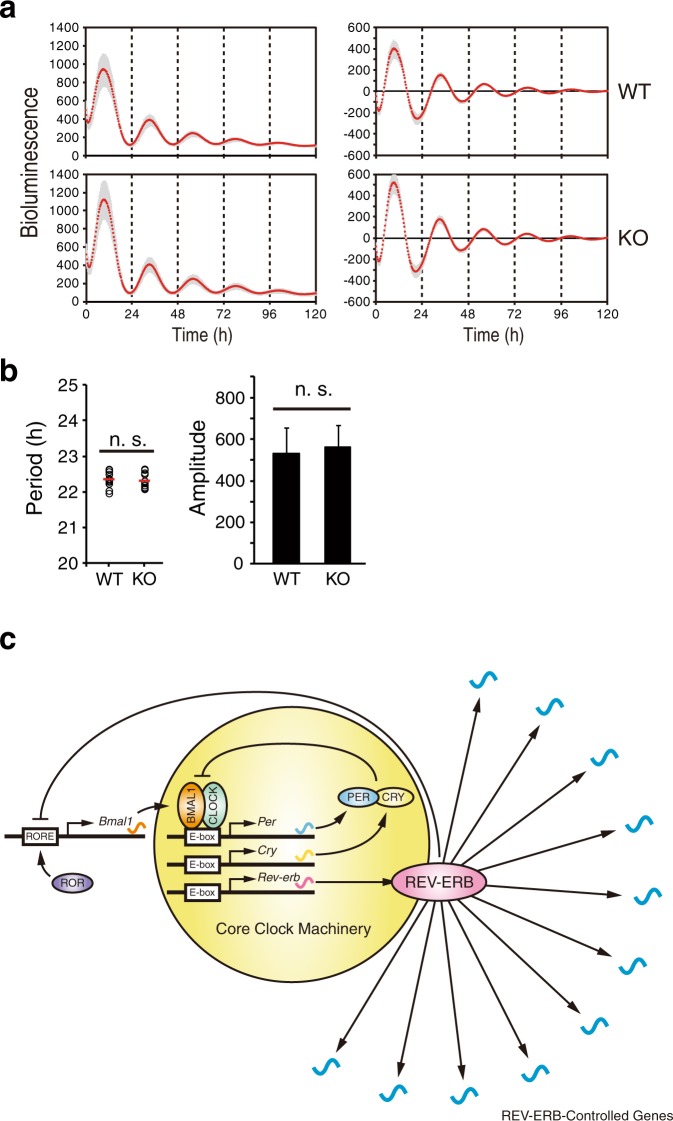
PER2^Luc^ bioluminescence rhythms in *Rev-erbα/β*-deficient cells. (**a**) PER2^Luc^ bioluminescence rhythms were measured in differentiated mES cells. Raw and detrended data are shown in the left and right graphs, respectively. The mean traces ± SD are plotted (n = 11 (WT) and 12 (KO)). (**b**) The period length and the amplitude are calculated and plotted. The values are the means ± SD (n.s., not significant). (**c**) Schematics of REV-ERB-mediated circadian gene regulation. REV-ERBs regulate the secondary loop of circadian transcriptional/translational feedback loops via RRE and control various output gene expression rhythms in a context-dependent manner.

To evaluate the impact of REV-ERBs deficiency on global gene expression rhythms, we performed a periodicity analysis on the time-course RNA-seq dataset. The number of cycling genes were 173 (1.2% of expressed genes) and 235 (1.6% of expressed genes) in WT and KO, respectively, and only 15 genes exhibited circadian oscillation of expression in both WT and KO cells (Fig. 3a, Supplementary Table S1). To our surprise, well-known circadian clock and clock-controlled genes (*Per2*, *Per3*, *Cry1*, *Rev-erbα*, *Rev-erbβ*, *Rorc*, *Dbp*, *Tef*, *Hlf*) were cycling in KO cells as well as WT cells. Furthermore, most of cycling genes were not common between WT and KO cells. This result indicates diverse changes in regulation of circadian output gene expression rhythms in KO cells, although the diverse phase distribution of cycling genes are similar in WT and KO cells with peaking around circadian time (CT) 18 (Fig. 3b,c). Transcription factor-binding motifs enrichment analysis revealed that HNF1 and NF-Y were enriched in the promoter of cycling genes in WT but not in KO (Fig. 3d, Supplementary Table S2), suggesting that HNF1 and NF-Y might be involved in the regulation of REV-ERB-dependent circadian output. This was supported by the results that *Hnf1b* was identified as a cycling gene only in WT cells (Supplementary Fig. S2), and that REV-ERBs bind to NF-Y and regulate target gene expression via the NF-Y-binding CCAAT motif in differentiating myoblasts^25^. Intriguingly, expression pattern of core clock and clock-controlled genes including *Per2*, *Per3*, *Cry1*, *Tef*, and *Hlf* were very similar between WT and KO cells (Fig. 3e). Peak expression levels of *Rev-erbα*, *Rev-erbβ*, and *Dbp* expression rhythms were elevated, while the basal expression of *Rorc* was reduced (Fig. 3e). In contrast to these sustained rhythms, *Bmal1* and *Npas2* expression rhythms are abrogated and constantly upregulated (Fig. 3f). The sustained rhythm of *Per2* and *Cry1* and constant upregulation of *Bmal1* and *Npas2* were confirmed by quantitative PCR analysis (Fig. 3g). Collectively, these results demonstrate that REV-ERBs repress *Bmal1* and *Npas2* mRNA expression but have only minor influence on mRNA expression rhythms of other core clock genes including *Per2*. These data suggest that REV-ERBs are not necessary for the circadian core clock gene expression rhythms but play an important role in regulation of circadian genetic program linking the circadian clock and output gene expression. Further analysis of the temporal expression profile of PER2^Luc^ bioluminescence in differentiated mES cells revealed that PER2^Luc^ bioluminescence rhythm in KO cells was as robust as that in WT cells (Fig. 4a). Both period length and amplitude were comparable between WT and KO cells (Fig. 4b). These results emphasize the importance of REV-ERBs as circadian clock mediators that regulate diverse output gene expression rhythms (Fig. 4c).

## Discussion

In this study, we revealed that *Rev-erbα/β* deficiency results in a drastic change in differentiation coupled formation of circadian network of gene expression, keeping core clock gene expression oscillating robustly. Accumulating evidence suggests that REV-ERBs regulate diverse physiological functions in cooperation with a variety of transcription factors in a context-dependent manner. It has been reported that REV-ERBs control expression of downstream genes involved in hepatic lipid metabolism via interaction with HNF6^15,26^, and that REV-ERBs regulate glucocorticoid action by binding to glucocorticoid receptor in liver^27,28^. A previous study has also demonstrated that REV-ERBα binds to NF-Y and regulates downstream gene expression via the CCAAT motif^25^. The cooperative transcriptional regulation by REV-ERBα and NF-Y may explain that CCAAT motif are enriched in promoter regions of cycling genes in WT cells but not in *Rev-erbα/β* KO cells. Also, REV-ERB-dependent circadian expression of *Hnf1b*, which was observed only in WT cells, is a possible mechanism describing the differential regulation of gene expression between WT and *Rev-erbα/β* KO cells. Such a drastic difference of cycling genes observed in the present study may suggest diverse regulation of circadian gene expression by REV-ERBs and co-regulating transcription factors. It has been demonstrated that different sets of genes are rhythmic in different tissues and/or cell types^7,29–31^. Thus, difference in cycling genes between WT and *Rev-erbα/β* KO cells may also reflect, at least in part, difference in cell types included in each cell population, although both WT and *Rev-erbα/β* KO mES cells differentiate into three germ layers. A heterogeneity of differentiated ES cell population can complicate the functional evaluation of changes in global gene expression rhythms. Given the pluripotency of ES cells, directed differentiation of ES cells into specific types of cells should help more systemized cell-based analysis of a wide range of tissue-specific REV-ERB functions. Taking into consideration the difficulties in analysis of mice with global knockout of both *Rev-erbα* and *Rev-erbβ*^8^, *Rev-erbα/β* double knockout ES cell model may provide a gateway to understand their *in vivo* circadian and physiological functions and to dissect their essential roles in both central and peripheral tissues. As a future theme, it is also intriguing to know the extent of functional redundancy and target gene specificity of REV-ERBα and REV-ERBβ by analyzing gene expression rhythms in *Rev-erbα* or *Rev-erbβ* single knockout cells.

Our results indicate that REV-ERBs are not essential for robust oscillation of core clock gene expression including *Per2*, while expression rhythms of the RORE-regulated genes such as *Bmal1* and *Npas2* were influenced by REV-ERBs. These data are consistent with the findings that *Bmal1* expression rhythms are not required for *Per2* expression rhythms. Previous studies have demonstrated that *Bmal1* but not *Per2* expression rhythms were disrupted on REV-ERBα and/or REV-ERBβ deficiency and that constitutive expression of *Bmal1* in *Bmal1*-deficient fibroblasts can restore *Per2* expression rhythms^6,32,33^. On the other hand, in *Rev-erbα/β* conditional double knockout mice, severe defects were observed in both hepatic circadian gene expression and behavioral rhythms^8^. It has also been shown that REV-ERBs bind to core clock gene loci including *Per2* and *Cry1*, suggesting direct regulation of these genes by REV-ERBs^8^. Given that REV-ERBs collaborate with various tissue-specific transcription factors and REV-ERB cistrome differs among tissues^15^, the extent to which REV-ERBs contribute to the core clock ticking may also be cell-type and context dependent. Nevertheless, our results indicate that REV-ERBs are not always essential for keeping the core circadian transcriptional/translational feedback loop.

In conclusion, we have established *Rev-erbα* and *Rev-erbβ* double knockout mES cells that develop the cell-autonomous circadian clock upon *in vitro* differentiation. The robust rhythms of PER2 expression and drastic change in a circadian gene expression network in differentiated *Rev-erbα/β* double-knockout ES cells suggest cell-autonomous functions of REV-ERBs as key factors of circadian output regulation. The established REV-ERBα/β deficient mES cell model provide a useful tool for examining REV-ERB-mediated circadian physiology in a cell-based assay system.

## Methods

### Cell culture

Mouse ES cells derived from *PER2*^*Luc*^ knock-in mice are described previously^19–21^. ES cells were maintained on MEF feeder cells in an ES medium (Glasgow Minimum essential Medium supplemented with 15% FBS (Hyclone), 0.1 mM MEM nonessential amino acids, 0.1 mM 2-mercaptoethanol, 1,000 U/mL of Leukemia inhibitory factor (LIF), and 100 U/mL penicillin-streptomycin).

### Plasmids

Human codon-optimized Cas9 expression vectors and sgRNA vectors were described previously^34^. A pair of oligos for the sgRNA targeting site was annealed and ligated into the sgRNA plasmid. The sgRNA target sites in introns adjacent to the targeted exon were determined by using CRISPRdirect^35^. The CRISPR-targeted sequences are as follows: Rev-erbα-T1, 5′-CCCAGACGTAGTTGATAGAGTT-3′; Rev-erbα-T2, 5′-TGCAAGGTGAGGCGGGTTAGGG-3′; Rev-erbβ-T1, 5′-CTACTGATAGATACCAGTAAGG-3′; Rev-erbβ-T2, 5′-GGGTTTAAACTCACTATGGTGG-3′. Underlines indicate protospacer adjacent motif (PAM) sequences.

### Establishment of *Rev-erbα*/*Rev-erbβ* double-knockout mES cells

Mouse ESCs were co-transfected with an hCas9 expression vector, two sgRNA expression vectors targeting *Rev-erbβ*, and a plasmid with a puromycin selection marker using FuGENE HD (Promega). The cells were selected with 2 μg/mL of puromycin for two days and then passaged for mutant cloning. ESC colonies were picked and cultured to establish mutant cell lines. *Rev-erbα* was targeted in established *Rev-erbβ*-deficient mES cells. The genomic deletion and the exon skipping in mRNA were confirmed by sequencing analysis of genomic DNA and cDNA, respectively.

### Sequence analysis of genomic DNA and cDNA

Genomic DNA samples were extracted from mouse ESCs grown under feeder-free conditions. cDNA samples were synthesized using Moloney murine leukemia virus (M-MLV) reverse transcriptase (Invitrogen) from total RNA extracted from mES cells using the RNeasy Mini kit (Qiagen) according to the manufacturer’s instructions. PCR was performed with PrimeSTAR MAX DNA Polymerase (TAKARA) under the following conditions: 98 °C for 1 min; 35 cycles of 98 °C for 10 sec, 60 °C for 5 sec, 72 °C for 10 sec; 72 °C for 20 sec; hold at 4 °C. PCR products were treated with Exonuclease I (New England Biolabs) and shrimp alkaline phosphatase (TAKARA) at 37 °C for 30 min followed by inactivation at 95 °C for 10 min and used for sequencing analysis. Sequencing was performed with the same primers as used for the PCR. The primer sequences are listed in Supplementary Table S3.

### *In vitro* differentiation of mouse ESCs

The *in vitro* differentiation of ESCs was performed as described previously^18,20^. Briefly, in order to form embryoid bodies (EBs), 2 × 10^3^ of the dissociated ESCs were seeded in low-attachment 96-well plates in a differentiation medium (DMEM supplemented with 10% FBS, 1 mM sodium pyruvate, 0.1 mM non-essential amino acids, GlutaMax-I (Invitrogen), 0.1 mM 2-mercaptoethanol, and 100 U/mL penicillin-streptomycin). Two days later, the EBs were plated onto gelatin-coated 24-well plates and cultured for 26 days in a differentiation medium, which was exchanged every other day.

### Real-time quantitative PCR

Total RNA was extracted from cells with the RNeasy Mini kit (Qiagen) according to the manufacturer’s instructions and subjected to cDNA synthesis with random hexamer primers and M-MLV reverse transcriptase (Invitrogen) according to the manufacturer’s instructions. Real-time quantitative PCR was performed with iTaq Universal SYBR Green Supermix (Bio-Rad Laboratories) and StepOnePlus real-time PCR system (Applied Biosystems). The PCR primers for quantitative PCR are listed in Supplementary Table S3. Of note, primers for *Rev-erbα* and *Rev-erbβ* were designed in the CRISPR-targeted exons to evaluate the successful deletion.

### RNA-seq

At differentiation day 28, cells were treated with 100 nM dexamethasone and frozen at the indicated time points. Total RNA was extracted from cells with the RNeasy Mini kit (Qiagen) according to the manufacturer’s instructions. Total RNA from differentiated cells from three embryoid bodies were pooled and used for the analysis. PolyA RNA selection, library construction using TruSeq RNA Sample Prep Kit v2, and sequencing were performed by Macrogen Japan (Kyoto) using Illumina NovaSeq6000 with 101-bp paired-end reads according to the manufacturer’s instructions (Supplementary Table S4). After adaptor sequence trimming using Trimmomatic^36^, sequence reads were mapped to the mouse genome (GRCm38/mm10) using STAR^37^. To obtain reliable alignments, the reads with a mapping quality of less than 10 were removed by SAMtools^38^. The University of California, Santa Cruz (UCSC) known canonical gene set (32,989 genes) was used for annotation, and the reads mapped to the exons were quantified using Homer^39^ as described previously^40^. Among the UCSC known canonical genes, genes were considered to be expressed if there were more than 0.5 reads per million reads mapped on average of 12 time points in the exons of the genes. RNA cycling was determined using Metacycle^41^ with P < 0.05, rAMP > 0.17, and the following options: minper = 20, maxper = 28, cycMethod = c(“ARS”,“JTK”,“LS”), analysisStrategy = “auto”, outputFile = TRUE, outIntegration = “both”, adjustPhase = “predictedPer”, combinePvalue = “fisher”, weightedPerPha = TRUE, ARSmle = “auto”, and ARSde- faultPer = 24. For transcription factor-binding motif analysis, known motifs in a range from −500 to + 500 of transcription start site (TSS) of cycling genes were discovered by Homer.

### Bioluminescence recording

For bioluminescence recording, the medium was replaced with a differentiation medium containing 0.2 mM luciferin and 100 nM dexamethasone. Bioluminescence was measured and integrated for one min at 20 min intervals with PMT-based equipment.

### Period and amplitude analysis

The bioluminescence data recorded by PMT were analyzed using a sine wave fitting. A linear baseline was subtracted from the raw data. The detrended data from between 36–108 hours was then used for analysis. Sine wave fitting was performed using the following equation:

$$y(t)=A{e}^{-kt}\,\sin (\frac{2\pi (t-\phi )}{\tau })$$

where *A* = amplitude, *k* = damping constant, *t* = time, *τ* = period, and *φ* = phase.

### Statistical analysis

For statistical analyses, two-tailed Student’s *t* tests were performed unless otherwise described.

## Supplementary information

Supplementary Figures

Supplementary Table S1

Supplementary Table S2

Supplementary Table S3

Supplementary Table S4

## Data Availability

The RNA-seq data has been deposited in the NCBI Gene Expression Omnibus (GEO) with the accession number GSE125696.
