# Supplementary material for: REV-ERBα and REV-ERBβ function as key factors regulating Mammalian Circadian Output
Source: Sci Rep. 2019 Jul 15;9:10171. doi: 10.1038/s41598-019-46656-0 (PMC6629614; doi:10.1038/s41598-019-46656-0)
Supplement: Supplementary file 1 — Supplementary Figures [file 41598_2019_46656_MOESM1_ESM.pdf]

**Supplementary information:**

# REV-ERBα and REV-ERBβ function as key factors regulating Mammalian Circadian Output

Ryosuke Ikeda[1,2], Yoshiki Tsuchiya[1,*], Nobuya Koike[1], Yasuhiro Umemura[1], Hitoshi Inokawa[1], Ryutaro Ono[1], Maho Inoue[1], Yuh Sasawaki[1], Tess Grieten[1], Naoki Okubo[2], Kazuya Ikoma[2], Hiroyoshi Fujiwara[2], Toshikazu Kubo[2], & Kazuhiro Yagita[1,*]

[1]Department of Physiology and Systems Bioscience, Kyoto Prefectural University of Medicine, Kawaramachi-Hirokoji, Kamigyo-ku, Kyoto, 602-8566, Japan.
[2]Department of Orthopaedics, Kyoto Prefectural University of Medicine, Kawaramachi-Hirokoji, Kamigyo-ku, Kyoto, 602-8566, Japan.

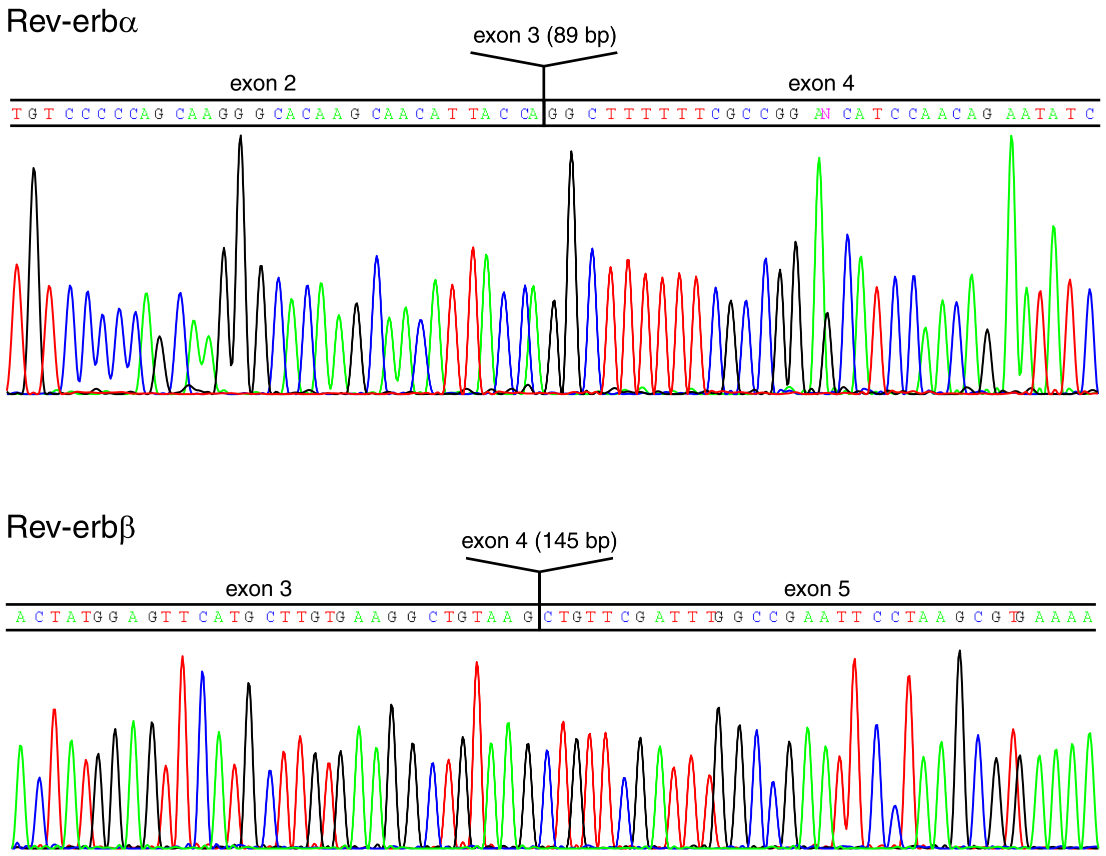


**Supplementary Fig. S1** Sequence of mRNA demonstrates a deletion of the targeted exon. cDNA was synthesized from total RNA extracted from WT and KO cells and CRISPR-targeted regions were amplified by PCR and sequenced.

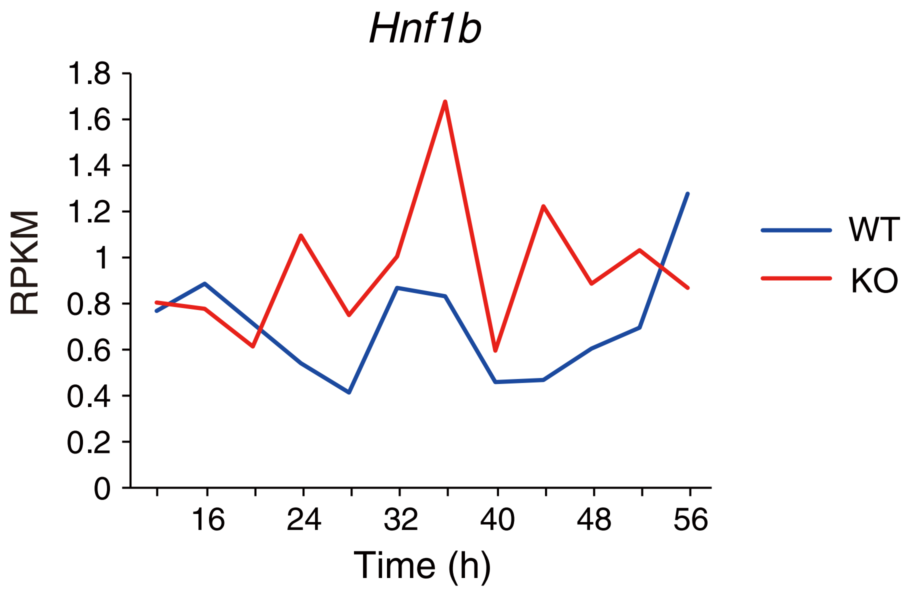


**Supplementary Fig. S2** Expression rhythms of *Hnf1b*. mRNA expression levels of *Hnf1b* in WT and KO cells are plotted with blue and red lines, respectively. *Hnf1b* is cycling in WT but not in KO.
